# Molecular Correlates and Recent Advancements in the Diagnosis and Screening of *FMR1*-Related Disorders

**DOI:** 10.3390/genes7100087

**Published:** 2016-10-14

**Authors:** Indhu-Shree Rajan-Babu, Samuel S. Chong

**Affiliations:** 1 Department of Pediatrics, Yong Loo Lin School of Medicine, National University of Singapore, Singapore 119074, Singapore; indhushree_rb@u.nus.edu; 2 Khoo Teck Puat—National University Children’s Medical Institute, National University Health System, Singapore 119228, Singapore; 3 Department of Laboratory Medicine, National University Hospital, Singapore 119074, Singapore

**Keywords:** fragile X syndrome, FXPOI, FXTAS, *FMR1*, AGG interruption, triplet-primed PCR, screening, CGG repeat, melting curve analysis, methylation

## Abstract

Fragile X syndrome (FXS) is the most common monogenic cause of intellectual disability and autism. Molecular diagnostic testing of FXS and related disorders (fragile X-associated primary ovarian insufficiency (FXPOI) and fragile X-associated tremor/ataxia syndrome (FXTAS)) relies on a combination of polymerase chain reaction (PCR) and Southern blot (SB) for the fragile X mental retardation 1 (*FMR1*) CGG-repeat expansion and methylation analyses. Recent advancements in PCR-based technologies have enabled the characterization of the complete spectrum of CGG-repeat mutation, with or without methylation assessment, and, as a result, have reduced our reliance on the labor- and time-intensive SB, which is the gold standard FXS diagnostic test. The newer and more robust triplet-primed PCR or TP-PCR assays allow the mapping of AGG interruptions and enable the predictive analysis of the risks of unstable CGG expansion during mother-to-child transmission. In this review, we have summarized the correlation between several molecular elements, including CGG-repeat size, methylation, mosaicism and skewed X-chromosome inactivation, and the extent of clinical involvement in patients with *FMR1*-related disorders, and reviewed key developments in PCR-based methodologies for the molecular diagnosis of FXS, FXTAS and FXPOI, and large-scale (CGG)_n_ expansion screening in newborns, women of reproductive age and high-risk populations.

## 1. Fragile X Syndrome

Partial or complete loss-of-function mutations in the fragile X mental retardation 1 (*FMR1*, OMIM 309550) gene, mapped to Xq27.3, cause fragile X syndrome (FXS, OMIM 300624). Most FXS-affected individuals (>99%) harbor a hypermethylated CGG-repeat stretch in the 5′-untranslated region of the *FMR1* exon 1 [1]. These allelic variants containing more than 200 CGGs—referred to as full-mutation (FM)—trigger aberrant methylation and heterochromatization of the *FMR1* promoter region, the epigenetic consequences that ultimately block *FMR1* gene expression [2]. Absence of the *FMR1*-encoded fragile X mental retardation protein (FMRP), which is critical for neuronal development and synaptic plasticity [3], results in the manifestation of FXS. Rare deletions and point mutations that could result in FMRP deficiency have been reported in fewer than 1% of FXS cases [4,5,6,7,8,9]. With an estimated prevalence of ~1 in 5000–8000 females and ~1 in 4000 males [10,11], FXS is the leading monogenic cause of intellectual disability (ID) and autism spectrum disorder (ASD) [12].

## 2. Molecular Determinants of FXS

FXS-affected individuals display a spectrum of neurologic, psychiatric and developmental issues, and additional ophthalmologic and facial characteristics, which often manifest with less severity in FM females as a result of X-chromosome inactivation (XCI) or Lyonization [13]. While most males with a FM have FXS, only 50% of FM females are clinically affected [14,15]. Intelligence quotient (IQ) in FM females is partly influenced by the XCI pattern; higher IQ levels have been reported in FM females who predominantly carry the FMRP-producing normal (NL, 5–44 CGGs) *FMR1* allele on the active X chromosome [16].

CGG-repeat size and methylation mosaicism can also have a positive effect on the cognitive functions of individuals with an *FMR1* FM and reduce the penetrance of FXS [17]. About 17%–41% of FM males have been identified as “size mosaics” who carry the transcriptionally active smaller premutation (PM, 55–200 CGGs) expansion in a proportion of their cells [18,19]. In addition, mosaicism for functional *FMR1* alleles in the NL repeat size range has been reported in ~1% of the FXS males [14]. In FM mosaics partial expression of the unmethylated PM/NL *FMR1* allele accounts for the milder cognitive involvement and less severe clinical presentation of FXS. However, mosaicism for a PM allele, which is usually associated with ~2–10-fold higher *FMR1* mRNA and slightly lower FMRP levels [20], can increase the risks of developing psychotic symptoms [21] and a late-onset neurodegenerative disorder, fragile X-associated tremor/ataxia syndrome (FXTAS, OMIM 300623) [22]. Methylation mosaicism, the presence of both fully methylated and unmethylated FM alleles, as opposed to the presence of methylated FM alone has been associated with better clinical outcomes and higher IQ levels in FXS individuals [17], and the subset of these methylation mosaics and unmethylated FM males who display moderate or normal phenotypes are referred to as high-functioning fragile X males [23,24,25]. Although the presence of an unmethylated FM could mean that FMRP is still produced, albeit at a lower amount due to the inefficient translation of the *FMR1* transcripts containing a hyperexpanded CGG-repeat stretch [26], the *FMR1* mRNA levels are generally higher in individuals with an unmethylated FM [25], which increases their risk of developing FXTAS [27,28,29]. The complexity in the phenotypic presentation of FXS can also be attributed to inter- and intra-tissue differences in *FMR1* CGG-repeat size and its extent of methylation [17]. In general, mosaicism for CGG allele size and degree of *FMR1* methylation can act as potential prognostic indicators of FXS.

## 3. Molecular Determinants of FXTAS

*FMR1* PM, although not responsible for FXS, predisposes ~40%–45% of the male [30,31] and ~8%–16% of the female carriers [31,32] to FXTAS, a condition that is characterized by intention tremor, cerebellar gait ataxia, peripheral neuropathy, parkinsonism, memory/cognitive function deficits and other psychological issues [33,34]. Age-dependent penetrance of FXTAS has been noted in both male and female PM carriers [31], with higher risks reported among individuals aged 70–79 years [31] and ≥80 years [30]. The risk and age-of-onset of FXTAS symptoms are also influenced by *FMR1* CGG-repeat length [35,36,37]. In addition, the neuropathological hallmark of FXTAS, i.e., numbers of intranuclear inclusions in neurons and astrocytes of affected individuals are associated with the number of CGG triplets, which highlights the clinical utility of repeat size analysis in predicting the extent of neurological involvement in PM carriers [38]. The penetrance of FXTAS is generally low in PM females, with the severity of clinical symptoms being directly proportional to the extent of skewed XCI of the NL *FMR1* allele [39,40,41,42].

Protein and RNA gain-of-function mechanisms have been proposed to underlie FXTAS disease pathogenesis [43]. Repeat Associated Non-AUG (RAN) translation of expanded *FMR1* CGG mRNA produces a toxic polyglycine-containing protein, FMRpolyG, which drives formation of intranuclear inclusions in FXTAS patient brains [43]. RNA toxicity results from *FMR1* over-expression, and the levels of *FMR1* mRNA are directly related to the number of CGG repeats in the PM allele [44]; an increase in *FMR1* mRNA of higher orders of magnitude has been reported in PM males carrying larger alleles of ~100–200 CGG repeats [20]. In female carriers, the increase in *FMR1* mRNA levels is linear in PMs of up to ~100 CGG repeats, beyond which a substantial increase in *FMR1* transcript expression was evident upon adjustment for X-inactivation ratio [45]. FMRP levels, on the other hand, are inversely correlated with CGG-repeat numbers especially in the higher PM size range [46]. While PM expansions are generally unmethylated, alleles at the upper end of the PM spectrum can be occasionally methylated in a small percentage of cells. Therefore, individuals with a large PM are more likely to display a greater degree of clinical involvement, possibly due to protein/RNA toxicity and/or reduction in FMRP caused by methylation and/or inefficient translation [46]. Inter- and intra-tissue differences in methylation and CGG-repeat size of the *FMR1* PM allele have also been noted [46].

## 4. Molecular Determinants of FXPOI

Approximately 20% of female PM carriers develop fragile X-associated primary ovarian insufficiency (FXPOI) [47], which causes infertility and early onset of menopause. The pathobiology of FXPOI still remains unclear, but a recent study reports detection of FMRpolyG-positive inclusions in the ovarian stromal cells of a subject with FXPOI, suggesting a possible role for RAN translation in FXPOI pathogenesis [48].

Repeat length and skewed XCI have been investigated as key determinants of FXPOI penetrance in PM women. The association between CGG-repeat size and risk for early onset of FXPOI symptoms is non-linear [49,50,51], with women carrying a mid-sized PM of 80–100 CGGs reported to have a greater risk for menopause at 40 years of age (odds ratio = 12.6; 95% confidence interval or CI, 5.3–30). In addition, the mean age at menopause in this group was lower compared to women with a PM of 59–79 CGGs or >100 CGGs [50]. The mid-sized PM cohort is also at increased risk for irregular menstrual cycles, subfertility and dizygotic twinning [51]. Particularly, the XCI pattern of PM does not appear to modify the risk for FXPOI [52,53,54,55].

## 5. Repeat Instability of PM-Sized Alleles

PM alleles show maternal bias for FM expansion during intergenerational transmission, and this risk for FM transition is governed primarily by maternal PM allele size [56,57,58,59,60,61]. Nolin et al. [60] reported that the risk of FM expansion increases with CGG-repeat length: 3.7%, 5.3%, 31.1%, 57.8%, 80.1% and ~100% of maternal alleles of 55–59, 60–69, 70–79, 80–89, 90–99 and 100–199 repeats, respectively, have been reported to undergo FM expansion, after correction for ascertainment. A subsequent study also confirmed the association between PM allele length and FM expansion risk [61]. Unstable allelic transmissions are also related to the lower density of repeat-stabilizing AGG anchors located at the 5′ end of the *FMR1* CGG-repeat region [62,63,64,65,66]. Uninterrupted CGG tracts are thought to mediate allelic instability via the formation of stable, hair-pin structures that promote strand slippage during replication. In contrast, the AGG triplets in interrupted CGG tracts act as “molecular brakes” and curb repeat expansions by destabilizing the secondary structures [67]. A higher degree of instability has been reported among alleles with long, uninterrupted CGGs at its 3′ end, with the instability threshold established at 34–38 pure CGGs [66]. Small PM alleles carrying 56 and 59 uninterrupted CGG repeats have expanded into a FM allele [60,68]. AGG information may help predict the risk of expansion of alleles with <100 CGG repeats [62], although additional studies are needed to better define the clinical utility of AGG mapping [11]. Thus, maternal *FMR1* CGG-repeat sizing and AGG mapping may both be important to obtain accurate risk estimates that facilitate predicting the likelihood of unstable transmission of *FMR1* alleles across generations.

## 6. Prevalence and Other PM-Associated Phenotypes

PM alleles are common in the general population, with variations in prevalence rates observed between different populations [1,11]: about 1 in 148 to 209 females and 1 in 290 to 430 males have been identified as PM carriers [69,70,71]. The phenotypic spectrum of the *FMR1* PM expands beyond the commonly reported FXTAS and FXPOI to include suggested association with anxiety, attention deficit hyperactivity disorder (ADHD), ASD, and developmental delay [72,73], and other medical co-morbidities such as hypertension [32,74,75], fibromyalgia [32,76,77], neuropathy [32], thyroid dysfunction [32,75,76], sleep apnea [78], migraine headaches [79] and seizures [32]. These phenotypes have been observed mostly in patients with FXTAS, although further research is necessary to confirm these disease associations. The number of co-occurring phenotypic features in PM carriers depends on the allele size; the larger the allele, the higher the number of co-occurring conditions [46].

## 7. Potential Risks and Instability of Intermediate-Sized Alleles

Intermediate/IM-sized alleles, also identified as gray zone-sized alleles, have 45–54 CGG repeats. Individuals with IM-sized allele do not exhibit any of the clinical symptoms of FXS, although they have been suggested to be at an increased risk for the PM-related FXTAS [80,81] and FXPOI [82,83,84]. However, a large-scale study on subjects with FXPOI failed to confirm this association [85], and the need to establish the role of IM alleles in FXTAS through larger studies has also been highlighted [11]. IM alleles also share the molecular characteristics of PM expansions; elevated *FMR1* mRNA and reduction in FMRP levels have been noted [44,86]. IM allele frequency in the general population varies between 1 in 22 to 66 females and 1 in 42 to 112 males [87].

About 16% of the maternal IM transmissions may result in a minor repeat size variation of one or two CGG(s) in the subsequent generation [1]. In general, alleles in the 50–54 repeat size range have a substantially higher risk for unstable transmission compared to alleles in the 45–49 repeat size range [88]. In addition, alleles lacking AGG interruptions have been reported to exhibit a greater degree of instability as opposed to alleles containing one or more AGG interruptions [65]. Notably, the offspring of women with an IM are not likely to be fragile X-affected, although they are at risk of inheriting a PM, which in turn can expand into a FM in a later generation. Studies have reported the expansion of an IM allele into FM in two generations [68,89,90]. Thus, the magnitude of CGG-repeat size change and the consequent risk for FM expansion in later generation(s) may be ascertained from the maternal IM allele length and number of AGG interruptions [14]. Accurate determination of CGG-repeat size and AGG numbers may both be important for the genetic counseling of at-risk individuals with 50–54 CGG repeats, although the impact of routinely incorporating AGG information into genetic counseling practice remains to be demonstrated [91]. The European Molecular Genetics Quality Network (EMQN) recommends that genetic counseling be offered to family members of IM individuals with 50–54 CGG repeats so as to facilitate the identification of relatives who may carry a PM and yet remain unaware of the associated risks [14].

## 8. Recommendation for *FMR1* Diagnostic Testing

The fragile X testing recommendation from the American College of Medical Genetics and Genomics (ACMG) is to genotype: (i) individuals with ID, autism or developmental delay, particularly those who have other physical or behavioral characteristics suggestive of FXS, a family history of FXS, or relatives with undiagnosed ID; (ii) fetuses of known PM and FM women; and (iii) individuals with a family history of FXS or undiagnosed ID who seek reproductive counseling [92]. The ACMG also endorses carrier testing in women who experience ovarian failure before 40 years of age, and men and women with late-onset intention tremor and gait ataxia, especially if they have a family history of FXS, movement disorders, premature ovarian failure or relatives with undiagnosed ID [92].

## 9. Diagnostic Tools for Characterizing the Multiple Molecular Facets of *FMR1* Expansions

Comprehensive molecular characterization of *FMR1* alleles requires information on: (i) CGG-repeat size; (ii) length of uninterrupted CGG repeats and number of AGG interruptions at the 3′ and 5′ ends of *FMR1*, respectively; and (iii) methylation status, and mosaicism for CGG-repeat size and methylation. Generally, a combination of methods might be required to characterize these different facets of the *FMR1* expansion mutation [11,93].

The gold standard fragile X test, Southern blot (SB) analysis, allows concurrent detection of large CGG-repeat expansions and determination of its methylation status, although precise resolution of *FMR1* allelic variants and AGG-interruption mapping cannot be achieved using this tool. Alternatively, polymerase chain reaction (PCR)-based approaches can be relied upon to characterize CGG-repeat size and/or AGG-interruption patterns, and several methods that are capable of detecting the entire spectrum of *FMR1* expansions have now been developed [94,95,96,97,98,99]. Therefore, many diagnostic laboratories employ a first-tier, high-throughput PCR-based approach to exclude non-expansion carriers and reflex only samples with an expansion for confirmatory SB analysis, as testing all samples by SB can be both labor- and time-intensive.

### 9.1. Flanking or Repeat-Spanning PCR

**Figure 1:**
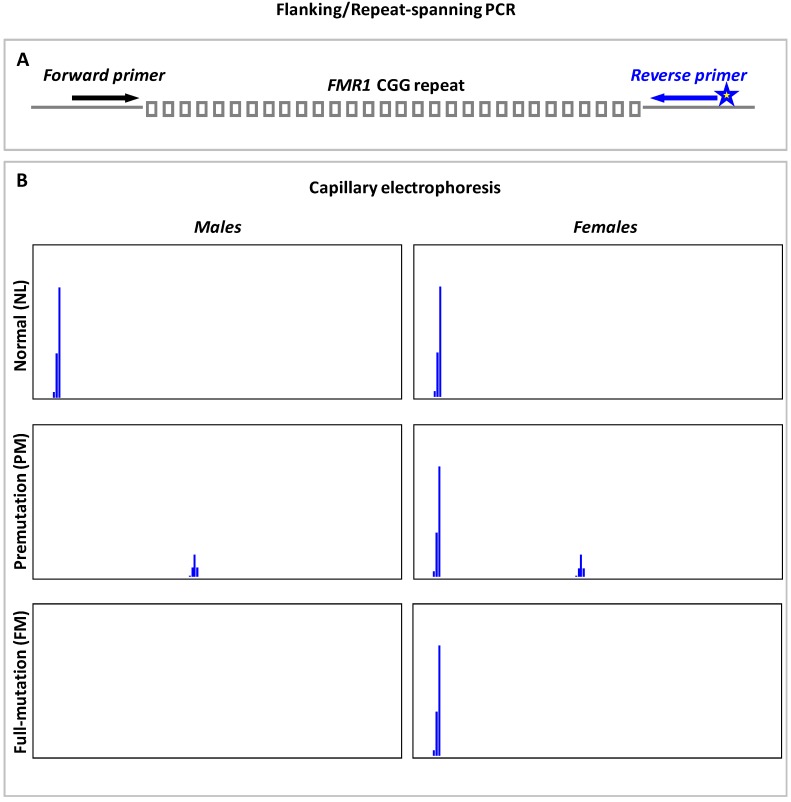
Flanking or repeat-spanning PCR: (**A**) Schematic depicting the flanking PCR primer design; and (**B**) expected capillary electrophoresis profiles of normal, premutation and full-mutation males and females.

The conventional flanking or repeat-spanning PCR technique uses two locus-specific primers to amplify across the *FMR1* CGG repeats. Schematic of flanking PCR, and expected capillary electrophoresis (CE) profiles of NL, PM and FM males and females are shown in Figure 1. Males with a NL or PM allele will yield an amplicon peak. Nonetheless, in males with a large FM, the flanking primers may fail to amplify across the CGG-repeat hyperexpansion and result in null-amplification. In general, flanking PCRs are known to perform less efficiently on PM and FM females due to the preferential amplification of NL allele. The homozygous NL females will yield a single amplicon peak, while females carrying a small, amplifiable PM will display a NL and a PM amplicon peak. However, in females with a large PM or FM, only the NL allele may get amplified. The detection of single NL peak in female samples, therefore, creates ambiguity as to whether they are homozygous NLs with two alleles of identical CGG-repeat size or heterozygotes with a NL and a non-amplifiable PM/FM. Consequently, all homozygous NL females require additional PCR-based testing or SB analysis to resolve zygosity and exclude the presence of an expanded allele. In addition, preferential detection of the smaller allele in a mosaic individual has been reported [100], and such skewed amplification in PM:FM or NL:FM mosaics can result in incorrect genotyping and altered risk assessment for FXS, FXTAS and FXPOI [11].

Hitherto, several flanking PCR assays for fragile X molecular diagnosis have been developed [95,96,100,101,102,103,104,105,106,107,108,109,110,111]. The initial PCR methods that were coupled to amplicon detection by slab-gel electrophoresis [101,103] were then replaced by the more robust fluorescent PCRs and CE to promote better allelic resolution and precise CGG-repeat sizing [95,96,102,104,105,106,108,109,110]. Notably, some of these assays are limited to the analysis of samples with alleles in the NL to small PM (~100–130 CGG repeats) range [103,104,106,110], while the improved protocols that have incorporated the Expand Long Template PCR System (Roche Diagnostics), dimethyl sulfoxide (DMSO) or betaine, and the dGTP analogue, 7-deaza-dGTP are more sensitive and detect up to small FM expansions, although more reliably in males than in females [100,105,108].

We previously devised a methylation-specific- or ms-PCR approach to determine the repeat size and methylation status of *FMR1* alleles, to reduce the need to perform SB analysis [101,102]. This strategy includes a preliminary sodium bisulfite treatment, followed by two separate reactions—a non-met-PCR and a met-PCR to amplify across unmethylated and methylated *FMR1* CGG repeats, respectively. Although efficient at sizing and determining the methylation status of *FMR1* alleles in the NL to PM range in samples of both sexes, met-PCR could not amplify methylated FM expansions [102].

Filipovic-Sadic et al. [96] described a flanking PCR tool that allows the detection of alleles of up to at least 1300 CGG repeats, in addition to resolving zygosity in heterozygous FM females and detecting FM mosaicism down to 1%. The amplicons when resolved by agarose gel electrophoresis (AGE) permits sizing of FM hyperexpansions, while CE provides single-repeat resolution and precise quantification of CGG repeats in NL, IM and PM alleles. The mPCR-CE [95] method was proposed for methylation and repeat-size assessment in males and females. Alleles of up to ~1000 CGG repeats can be readily detected, while simultaneously quantifying their degree of methylation. Grasso et al. [112] validated the mPCR-CE approach, and reported better allelic size resolution and 100% sensitivity in detecting FM alleles, higher sensitivity in detecting mosaicism for repeat size and methylation, and detection of FM mosaicism down to 1%. In conjunction with triplet repeat primed PCR (Please refer to Section 9.3 for assay design) mPCR-CE can eliminate the need for SB analysis in a clinical setting. Similar to the Filipovic-Sadic CE analysis, the mPCR-CE also precisely sizes NL, IM and PM alleles, but does not resolve FMs that are >250 CGG repeats. While FMs of ~1000 to 1300 repeats are claimed to be detected by both methods, the upper limit of expansion that can be detected is contentious.

Flanking PCRs also do not facilitate AGG-interruption mapping. Another issue with flanking PCRs is the difference in the expected and observed CGG-repeat sizes of *FMR1* alleles, conceivably owing to the increased electrophoretic mobility of the GC-rich *FMR1* amplicon fragments relative to DNA size standards [113,114,115], which could result in incorrect genotypic classification of alleles with repeat sizes bordering between two allele classes. It appears that the relationship between amplicon base pairs and CGG-repeat size is non-linear, and to prevent erroneous *FMR1* genotyping it is necessary to determine repeat size of tested samples with reference to a standard curve generated from an allelic ladder of sequence-verified control DNAs or validated reference materials from the Coriell Cell Repositories (Camden, NJ, USA), the National Institute for Biological Standards and Controls (UK) or the National Institute of Standards and Technology (Gaithersburg, MD, USA), etc., as recommended by the EMQN fragile X testing guidelines [14].

### 9.2. PCR-Based Assays for FMR1 Regulatory Region Methylation Analysis

Fragile X testing methods that analyze the methylation status of CpG dinucleotides in the *FMR1* promoter region have been proposed [116,117,118,119,120]. Typically, these procedures involve sodium bisulfite treatment of genomic DNA prior to PCR. Fragile X males are differentiated from NL/PM males based on the methylation-specific amplification of bisulfite-modified CpG sites; while the former are amplified by primers that target methylated CpGs, the latter show amplification only with primers that recognize unmethylated CpGs [116,118,119]. Unless the *FMR1* CGG-repeat length is known, NL and PM males, and size and methylation mosaic FM males cannot be discerned solely by promoter-based methylation methods. In addition, owing to XCI, amplicon profiles of NL females may appear similar to that of carrier and FXS-affected females, which could make fragile X diagnosis in most cases difficult, consequently limiting these assays to the detection of FM males only.

The Methylation-Specific Multiplex Ligation-Dependent Probe Amplification or MS-MLPA assays, involving numerous oligonucleotide probes complimentary to the *FMR1* target sequence that spans a methylation-sensitive restriction endonuclease site, is also available for the diagnosis of FXS in males [121,122]. MS-MLPA generates signals depending on the *FMR1* methylation status; briefly, enzymatic digestion and PCR of hybridized and ligated MS-MLPA probe/DNA complexes result in the successful amplification of methylated regions, while probes juxtaposed to unmethylated sites are digested and no PCR signal is generated. Though unmethylated NL/PM and methylated FM males are effectively discriminated, MS-MLPA cannot be relied upon for *FMR1* genotype analysis in female samples.

An alternative to analysis of the classical *FMR1* CpG island involves methylation analysis of FREE (Fragile X Related Epigenetic Elements) DNA on a MALDI-TOF mass spectrometry (MS)–based EpiTYPER instrument [123]. This method has been somewhat superseded by a real-time PCR based MS-QMA (Methylation-Specific-Quantitative Melt Analysis) quantitation of the FREE2 CpG methylation [124], whereby degree of FREE2 methylation is inversely correlated with FMRP expression in males and females carrying an expanded CGG allele [123,125]. MS-QMA does not identify all FM individuals, but instead detects low-functioning (IQ < 70) FXS males and females with high sensitivity [124]. However, predicting cognitive function based on MS-QMA methylation ratio (MR) can be complicated in female samples as some normal controls and a significant proportion of PM/FM females with IQ > 70 appear to yield MRs that overlap with that of, or fall within the range of, low-functioning FM females, or yield MRs in the borderline range. Therefore, in the diagnostic setting, MS-QMA must be accompanied by a second-tier CGG-repeat size analysis method to resolve the overlapping *FMR1* allelic classes.

### 9.3. Triplet Repeat Primed PCR

Triplet Repeat Primed PCR (TRP-PCR or TP-PCR) was first proposed by Warner et al. [126] for detecting CAG-repeat expansions that cause myotonic dystrophy. The unique primer design of the TP-PCR assay makes it the most ideal tool for detecting trinucleotide repeat expansions. As such, three oligonucleotide primers are employed, including a fluorescently tagged locus-specific primer that hybridizes to the region immediately upstream or downstream of the repeats, a triplet-primed (TP) primer that anneals randomly at multiple positions within the repeats and generates a mixture of distinctly-sized amplicon fragments, which are then amplified to a detectable intensity by a third Tail primer that bears the same sequence as the 5′ overhanging stretch of TP primer [126]. The heterogeneous TP-PCR amplicons can be visualized as “smears” on agarose gels or as “ladders or stutters” of fluorescent peaks (that differ from each other by one repeat) in the CE electropherograms.

**Figure 2:**
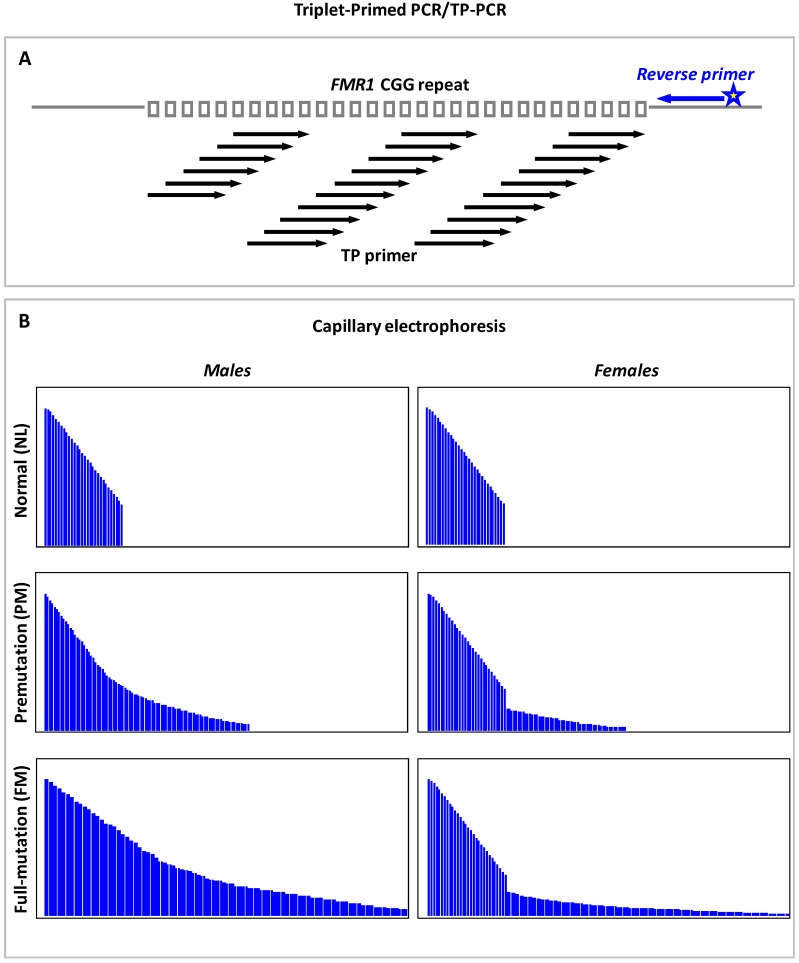
Triplet-Primed PCR or TP-PCR: (**A**) Schematic depicting the TP-PCR primer design; and (**B**) expected capillary electrophoresis profiles of normal, premutation and full-mutation males and females. For simplification, the Tail primer is not shown.

Figure 2 presents the schematic of TP-PCR primer design, and the expected CE profiles of NL, PM and FM males and females. Generation of continuous, uninterrupted amplicon peaks would signify the absence of AGG interruptions, and CGG-repeat size is determined from the number of amplicon peaks. In contrast to flanking PCR, TP-PCRs will reliably detect expansions in both males and females, and also enable the differentiation of homozygous NL and heterozygous FM females.

The Warner et al. TP-PCR approach has now been modified for successful amplification and detection of CGG-repeat expansions in the *FMR1* gene [94,97,98,99,101,102,127]. We initially developed a methylated allele TP-PCR (mTP-PCR) to detect all methylated expansions; however, although FM males can be readily identified from the mere detection of mTP-PCR amplicon peak “stutters”, females with methylated PM cannot not be differentiated from those with a FM by mTP-PCR alone [101,102]. The Lyon et al. [127] *FMR1* TRP-PCR method for expansion screening can detect all PM and FM males and females but does not discriminate between them, while the Hantash et al. [97] method can amplify across the full range of mutation in both sexes. The above-mentioned methods follow the Warner et al. assay design in most aspects, with a slight difference in the Hantash et al. method, which employs a modified reverse primer that hybridizes both at the junction of CGG repeats and within the repeats.

The CGG repeat primed *FMR1* PCR strategy of Chen et al. [94] uses two *FMR1* locus-specific primers flanking the repeats and a third primer complementary to the repeats. Full-length amplicons are generated by the flanking primers, while amplicon stutters are produced from the annealing of CGG primers. Besides detecting *FMR1* alleles that ranged from NL to FM in both males and females, the Chen et al. method was also found to be robust in spotting mosaicism for PM and FM down to 1% and 5%, respectively, in a high NL background [128]. We developed and validated the performance of a TP-PCR assay (direct TP-PCR CE) in precisely sizing NL, IM and PM alleles, and consistently amplifying beyond 200 CGG repeats in FM males and females, and also reported a FM detection sensitivity of 4% in the presence of 96% NL DNA [98]. Intriguingly, the priming pattern of the CGG primers used in these assays can reveal the number and distribution pattern of AGG interruptions within the CGG-repeat stretches of alleles in the NL, IM and PM size ranges [94,98]. It is also important to note that these assays verify FMs based on the detection of peak stutters that extend beyond the 200-repeat cutoff and a characteristic FM peak at ~1.1 kb, but do not determine the actual size of the FM expansions.

We also developed a methylation-specific TP-PCR (msTP-PCR) assay that determines the methylation status of *FMR1* alleles, in addition to CGG-repeat size and AGG-interruption analyses [99]. This single-tube duplex-PCR is performed on bisulfite-treated DNA, and includes two sets of primers for amplifying the methylated and unmethylated *FMR1* alleles simultaneously. Mosaicism for FM can be detected down to 5% in a PM background of 95%, and sensitivity in identifying PM:FM mosaics is higher relative to SB. The msTP-PCR is an all-inclusive test that characterizes all the molecular facets of *FMR1* expansions, and in a diagnostic setting has the potential to virtually eliminate the need for SB.

In general, the improved TP-PCR methodologies [94,98,99] address most of the limitations of flanking PCR-based tests. Owing to its ability to amplify from within the CGG-repeat region, these methods detect all FMs (irrespective of the actual length of the mutant *FMR1* allele), resolve zygosity issues in females, determine both number and position of AGG interruptions, and facilitate accurate sizing of NL to PM alleles. As the CGG-repeat sizes are determined by merely counting the number of detected amplicon peaks, TP-PCR-determined (CGG)_n_ is also more exact than repeat size derived from direct conversion of flanking PCR-amplified fragment lengths.

## 10. Population-Based Screening for *FMR1* Expansions

Implementation of universal newborn screening (NBS) for FXS, preconception and prenatal carrier screening have long been considered [10,72,129,130,131,132,133]. In 2006, the ACMG did not endorse the inclusion of FXS to universal NBS panel primarily due to the lack of evidence supporting the benefits of early diagnosis and the lack of a cost-effective screening tool [133]. It is now evident that early FXS diagnosis can facilitate timely interventions that could be beneficial [72], alleviate the “diagnostic odyssey” experienced by families and inform parents of their risks of having fragile X affected children through subsequent pregnancies, and also promote cascade testing of extended family members [129,134] and identification of relatives who harbor a PM and are likely to benefit from prophylactic interventions [33] and diagnosis-based management. In addition, several pilot studies have demonstrated the cost-effectiveness and feasibility of conducting large-scale NBS for FXS [69,135,136,137,138], and surveys on attitudes toward screening also reveal the growing support for fragile X NBS [129,139,140,141,142,143,144,145,146].

Identification of PM carriers through screening women of reproductive age can avoid the birth of FXS-affected children, and provide crucial information related to their risk for FXPOI, which can prompt them to make informed family planning decisions prior to fertility decline. Musci et al. [147] demonstrated that the prenatal population-based carrier screening is both feasible and cost-effective, and nationwide prenatal carrier screening is now being effectively implemented in Israel where the PM frequency is high [148,149], while preconception screening is mostly preferred by genetic health professionals [10,141,150].

## 11. *FMR1* Molecular Tests for Large-Scale Screening Applications

An ideal screening test should be rapid, high-throughput and easy to perform, while also being cost-effective, sensitive and specific. SB, which is laborious and low-throughput, is not the method of choice for large-scale screening. Alternative PCR-based *FMR1* tests have been established [97,98,127,151,152]. Primarily, these are flanking- and/or TP-PCR-based screening methods that are coupled to amplicon detection by AGE, CE or melting curve analysis (MCA).

In 2008, Tassone et al. [151] proposed a two-tier strategy for screening newborn and high-risk populations. The first round screen relies on flanking PCR, and is followed by a second round PCR—with CGG-annealing chimeric primers—when the samples tested are “suspected” as positive for *FMR1* expansion. Essentially, males who display null-amplification and females with a single band are verified using the chimeric primers. Though this sequential approach eventually minimizes the number of samples that need SB analysis, a significant proportion of NL females (~25%−40%) who are homozygous for *FMR1* alleles, and indistinguishable from heterozygous female samples carrying a large PM or FM allele and a NL allele, require reflexing to second-tier PCR for resolving zygosity. In addition, the first-tier flanking PCR and AGE may not allow for reliable categorization of samples with a borderline *FMR1* allele, and may not discriminate between two allelic bands in some heterozygous NL females. Although these issues can be resolved by CE that offers single-repeat resolution [69,153], screening costs will be increased [98], and sequence-verified controls will be needed to correct for mobility shift when sizing the *FMR1* amplicons. Regardless, the recommended approach of excluding from further analysis those males who generate a single band or peak, would result in the incorrect genotyping of NL:FM mosaics as NLs. Thus, it is critical to have a first-tier test that detects mosaicism for *FMR1* expansions efficiently, and generates fewer ambiguous screen results that may warrant further testing.

*FMR1* genotyping methods based on TRP-PCR/TP-PCR and CE have been proposed as first-tier tests for NBS and carrier screening [97,127]; however, these strategies still require post-PCR amplicon manipulations, which may be less than ideal for population based applications. To minimize the need for post-PCR manipulation, we developed a screening tool that involves MCA of the TP-PCR amplicons on a real-time PCR system [152]. MCA is ~15 times cheaper than CE [98]. Basically, the TP-PCR for MCA is performed with unlabeled primers in the presence of SYBR Green I Nucleic Acid Dye, whose dissociation from amplicon fragments at optimal melting temperature results in a sharp drop in the fluorescence [152]. This proof-of-concept study proposed a single-step, closed-tube method that functions on the principle of categorizing samples as “expanded” or “non-expanded” based on the temperature at which their melt profiles dropped to baseline or zero −d*F*/d*T* levels. The threshold or cutoff temperature for distinguishing NL/IM from expanded samples was established using a fragile X reference control sample of known CGG-repeat size.

This *FMR1* direct TP-PCR (dTP-PCR) MCA strategy involves two assays: a 5′ and a 3′ assay that each employs a *FMR1*-specific primer designed to anneal to the region immediately upstream or downstream of CGG repeat, respectively. Both assays are recommended to be performed as deletion at either end of a CGG-repeat expansion—reported in <1% of FXS cases—can prevent annealing of the *FMR1* locus-specific primer, and result in drop-out of the expanded allele and failure to detect a true positive, if either one of these assays was applied alone [152]. While both 5′ and 3′ assays are efficient in identifying the presence of an expansion, the 3′ assay was found to be more robust in generating distinct melt profiles for NL, IM and expanded samples [152,153], and has been applied as a first-tier screen to assess the prevalence rates of IM, PM and FM among the children attending special education institutions in Sri Lanka [154]. Genomic DNA isolated from buccal swabs were used for preliminary screening, and all identified “expansion positives” were correctly verified by confirmatory molecular diagnostic tests. Lyons et al. [153] and Lim et al. [155] have validated the performance of this screening tool, and reported specificities of 95.1% (95% CI, 87.8% to 98.6%) and 99.6% (95% CI, 98.5% to 99.9%), respectively, while both studies recorded a sensitivity of 100% in detecting expansions. In a high NL DNA background, the assay was able to spot mosaicism for PM and FM down to 7.5% and 20%, respectively [155].

**Figure 3:**
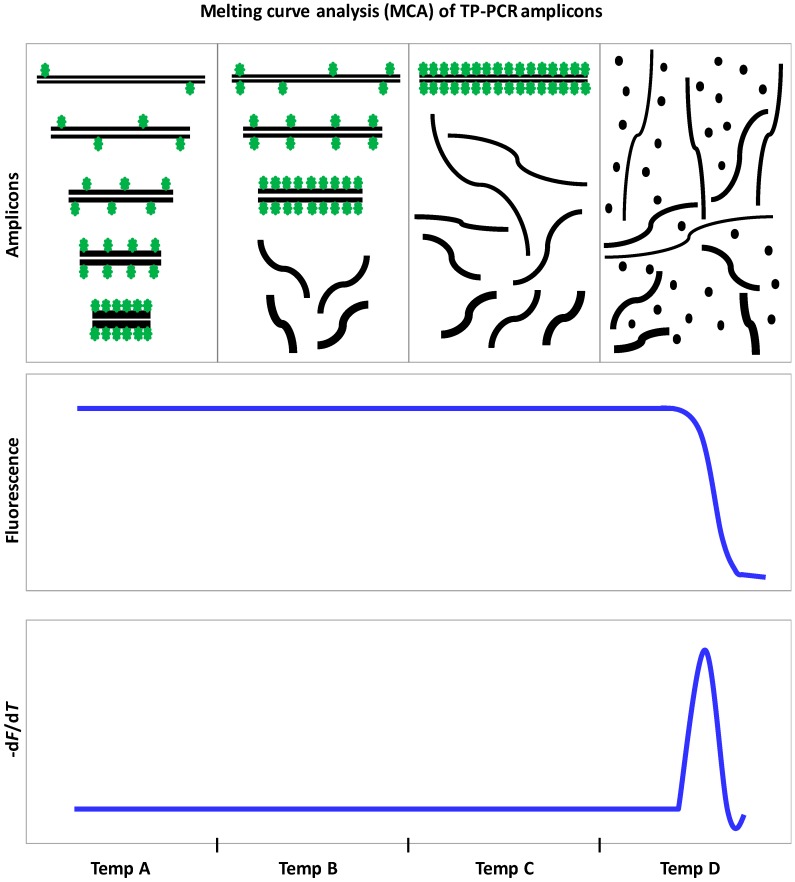
Melting curve analysis (MCA) of TP-PCR amplicons. Schematic illustrating how MCA of heterogeneous TP-PCR amplicons results in the generation of a single melt peak. Dissociation of double-stranded PCR products and re-distribution of SYBR Green I Dye from the shorter to larger fragments with gradually increasing temperature (Temp A to Temp C) is shown. At Temp D, when the largest amplicon strands dissociate, SYBR Green I is completely released and remains unbound to DNA, resulting in a sharp decrease in fluorescence. −d*F*/d*T*, first negative derivative of fluorescence versus temperature.

Recently, we developed an improved dTP-PCR MCA assay that produces sharper and more distinct melt peaks. This enabled the determination of melt peak temperature (T_m_) and increased the overall confidence in genotyping samples with a borderline *FMR1* allele [98]. In general, the T_m_ showed good correlation with CGG-repeat size, and in most cases, it reflected the temperature at which the largest fragments in the heterogeneous TP-PCR amplicon pool denatured. This is facilitated by re-distribution of the non-saturating SYBR Green I Dye molecules from shorter to larger amplicon fragments as the temperature is gradually increased, and eventually when the highest melting point or T_m_ is reached, the bound SYBR Green I molecules are released from the amplicons (Figure 3). We adopted a unique approach of defining an “indeterminate zone”, which is basically the melting temperature range of IM alleles, using the T_m_s of a 46-repeat and a 54-repeat fragile X reference control. The samples analyzed are then classified as NL, IM/indeterminate, or expanded depending on their T_m_s relative to the indeterminate zone. A significant improvement in the assay’s potential to detect mosaicism was also noted; FM as low as 1% could be identified in the presence of 99% NL DNA. Importantly, the prospect of employing fragile X reference control(s) of desired CGG-repeat length makes it easier to customize this screening tool for different purposes.

In a subsequent validation study [156], we characterized several key parameters that could influence the T_m_s and potentially result in an altered *FMR1* genotypic classification of a sample. With an ability to detect expansions from as little as 1 ng input DNA, using genomic DNA isolated from a wide variety of sources including buccal swabs, saliva, peripheral blood and dried blood spots, the dTP-PCR MCA screen is readily adaptable to different real-time PCR systems and is a cost-effective method that is both robust and ideal for ruling out *FMR1* expansions in a majority (>97%) of samples from the general population [70,71]. The dTP-PCR MCA can be complemented by test(s) for CGG-repeat size and/or methylation analysis to confirm the genotypes of identified “screen positives”.

Coffee et al. [157] proposed a PCR-based FXS screen (quantitative methylation-sensitive PCR or Q-MSP) for detecting and quantifying *FMR1* promoter methylation in newborn males. The Q-MSP displayed 100% sensitivity and specificity in discriminating unaffected males from those carrying a methylated FM. Excessive methylation was also evident in most FM females, although no correlation with FXS penetrance was observed. Elias et al. [158] had established a multiplex methylation-specific real-time PCR and MCA screen, which relies on the differences in the T_m_s of unmethylated and methylated *FMR1* gene promoter to genotype NL, PM, and FM males with or without mosaicism for a PM allele. In general, techniques that only evaluate the methylation of CpG dinucleotides in the CGG-repeat flanking sequence, without analyzing the CGG repeat itself, cannot distinguish NL/PM males from high-functioning males who harbor an unmethylated FM, and thus may not detect all fragile X cases [159]. In addition, FXS diagnosis in females is challenging due to the presence of both methylated and unmethylated NL and PM *FMR1* DNA arising from XCI.

We developed a dual 5′ and 3′ unmethylated TP-PCR (uTP-PCR) and methylated TP-PCR (mTP-PCR) and MCA screen for detecting PM and FM expansions and their methylation status in males and females [160]. With reference to the cutoff temperature established using a 54-repeat control, NL samples displayed low T_m_ uTP-PCR and/or mTP-PCR peaks, while samples with a methylated PM/FM displayed a higher T_m_ mTP-PCR peak. Males with PM or unmethylated FM are expected to generate uTP-PCR peaks that display higher T_m_ relative to the control, and size mosaic FM males can be readily detected by the presence of high T_m_ peaks in both uTP- and mTP-PCR panels.

Intriguingly, the XCI patterns in females can be discerned from their uTP- and mTP-PCR melt peak profiles. For instance, PM females who carry NL and PM allele equally on active and inactive X chromosomes will generate high T_m_ uTP- and mTP-PCR peaks, while PM females with skewed inactivation of the X chromosome that harbors the mutant allele will exhibit a low and a high T_m_ peak in the uTP- and mTP-PCR panels, respectively, and a reverse MCA pattern can be expected if skewing is in the direction of the NL allele. In FXS females, the FM allele is methylated on both the active and inactive X chromosomes. This then results in the generation of a melt profile pattern of low T_m_ uTP-PCR peak and high T_m_ mTP-PCR peak for samples with random X-inactivation or skewed inactivation of the X chromosome carrying the FM allele. Markedly, FM females with skewed inactivation of the X chromosome carrying the NL allele, who are likely to be penetrant for FXS, can be easily differentiated as these samples will only display a mTP-PCR amplicon peak of high T_m_, while no *FMR1* amplicon peak will be seen in the uTP-PCR panel. In NL:FM mixtures, FM mosaicism as low as 5% can be detected by mTP-PCR MCA. This tool has potential applications in voluntary NBS, and can also be applied to study the association between XCI and severity and/or age-of-onset of FXTAS/FXPOI in PM women.

## 12. FMRP Antibody Tests for FXS Diagnosis and Screening

Immunocytochemical assays employing specific antibodies to determine the presence/absence of FMRP in lymphocytes [161,162] and hair roots [163,164] have also been developed. Normal and FXS-affected males are differentiated by the presence and absence of FMRP-positive cells, respectively, but FMRP detection in FM females with skewed XCI of the expanded allele is likely to complicate FXS diagnosis in females [162]. Hair root FMRP analysis was reported to be highly reliable in predictive testing of FM females with cognitive impairment [164], perhaps due to the shared embryonic origin of skin/hair and neurons [165]. FMRP tests for the prenatal diagnosis of fragile X-affected male fetuses have also been developed [166,167,168]. Besides, FMRP tests can be used to investigate “expansion negative” cases who present clinical phenotypes suggestive of FXS [14]. FMRP immunoassays have also been proposed for large-scale population-based screenings [169,170,171]. Generally, these screens can detect patients with FXS and exclude FMRP-positive FM individuals who are less likely to have cognitive impairment, but they cannot be used to identify PM carriers.

## 13. Concluding Remarks

As discussed above, there are several PCR-based assays that can effectively discern the entire spectrum of *FMR1* CGG-repeat mutation and provide a reliable diagnosis in most cases with a FXS-related condition. While the current FXS testing recommendation is to always perform SB together with conventional PCR [11], it is also widely accepted to use a first-tier PCR test that enables CGG-repeat size and/or methylation analysis, and reserve SB only for those critical samples that require a confirmatory diagnosis. Most of the recently developed PCR assays excel in their ability to size alleles of up to a PM size range, and detect FM and low-level mosaicism for *FMR1* expansions and identify unique methylation patterns, and in some instances, even outperform the gold standard SB analysis. The performances of these diagnostic assays have been tested using verified fragile X reference DNA and further validated using patient DNA, and their limitations, sensitivity and specificity in detecting the presence of *FMR1* expansion have been investigated extensively. In addition, while the newer CGG-repeat analysis methods will yield a definitive diagnosis in most fragile X cases, the rarer non-CGG repeat mutations can be detected by sequencing and array-based technologies [4,172].

FMRP tests, especially those involving hair root analysis, have been proposed as an ideal fragile X screening tool [165]. Besides, MCA-based TP-PCR methods for *FMR1* molecular analysis also offer a simple and cost-effective solution for reliable and sensitive detection of “expansion positive” samples regardless of gender. Notably, the high-throughput MCA platform also minimizes sample mix-ups in large-scale screening settings by eliminating or minimizing post-PCR manipulations. However, several key issues related to predictive testing for the late-onset, PM-related FXTAS/FXPOI in newborns, identification of FM girls who may or may not develop the symptoms of FXS, and genetic counseling problems for the complex phenotypic spectrum of *FMR1*-related conditions, still remain to be fully addressed. Should FXS be endorsed for inclusion in universal newborn and carrier screening programs, the merits, limitations and utilities of the methods discussed in this review can assist in the selection of a tool that will suit the objectives of the screening program.
